# Supplementary material for: Larvicidal properties of terpenoid-based nanoemulsions against the dengue vector Aedes aegypti L. and their potential toxicity against non-target organism
Source: PLoS One. 2024 Feb 7;19(2):e0293124. doi: 10.1371/journal.pone.0293124 (PMC10849395; doi:10.1371/journal.pone.0293124)
Supplement: Attachment — Submitted filename: PONE-D-23-16634_rev.pdf [file pone.0293124.s002.pdf]

PLOS ONE

Larvicidal properties of terpenoid-based nanoemulsions against the Dengue vector Aedes aegypti L. and their potential toxicity against non-target organism

--Manuscript Draft--

**Manuscript Number:**	PONE-D-23-16634
**Article Type:**	Research Article
**Full Title:**	Larvicidal properties of terpenoid-based nanoemulsions against the Dengue vector Aedes aegypti L. and their potential toxicity against non-target organism
**Short Title:**	Terpenoid-based Nanoemulsions: Larvicidal Potential Against Aedes aegypti and Non-Target Organism Toxicity
**Corresponding Author:**	Vincent Corbel<br>Institut de recherche pour le developpement<br>Montpellier, FRANCE
**Keywords:**	Dengue; Aedes aegypti; larval control; terpenes; p-cymene; myrcene; nanoemulsion
**Abstract:**	The development of insecticide resistance in mosquitoes of public health importance has encouraged extensive research into innovative vector control methods. Terpenes are the largest among Plants Secondary Metabolites and have been increasingly studied for their potential as insecticidal control agents. Although promising, terpenes are insoluble in water, and they show low residual life which limits their application for vector control. In this study, we developed and evaluated the performances of terpenoid-based nanoemulsions (TNEs) containing myrcene and p-cymene against the dengue vector Aedes aegypti and investigated their potential toxicity against non-target organisms. Our results showed that myrcene and p-cymene showed moderate larvicidal activity against mosquito larvae compared to temephos an organophosphate widely used for mosquito control. However, we showed similar efficacy of TNEs against both susceptible and highly insecticide-resistant mosquitoes from French Guyana, hence suggesting an absence of cross-resistance with conventional insecticides. We also showed that TNEs remained effective for up to 45 days in laboratory conditions. The exposure of zebrafish to TNEs triggered behavioral changes in the fish at high doses but they did not alter the normal functioning of zebrafish organs, suggesting a good tolerability of non-target organisms to these molecules. Overall, this study provides new insights into the insecticidal properties and toxicity of terpenes and terpenoid-based formulations and confirms that TNE may offer interesting prospects for mosquito control as part of integrated vector management.
**Order of Authors:**	Jonatas Duarte
	Stéphane Duchon
	Leonardo Delello Di Filippo
	Marlus Chorilli
	Vincent Corbel
**Additional Information:**	
**Question**	**Response**
**Financial Disclosure**<br><br>Enter a financial disclosure statement that describes the sources of funding for the work included in this submission. Review the submission guidelines for detailed requirements. View published research articles from *PLOS ONE* for specific examples.	This project has received funding from Sao Paulo research foundation-FAPESP under the grants n°2021/11487-4 and 2019/25125-7. This project also receives a co-funding from the European Union HORIZON-MSCA-2021-SE-01 (INOVEC project), under the grant n°101086257.

Powered by Editorial Manager® and ProduXion Manager® from Aries Systems Corporation

This statement is required for submission and **will appear in the published article** if the submission is accepted. Please make sure it is accurate.<br><br>**Unfunded studies**<br>Enter: *The author(s) received no specific funding for this work.*<br><br>**Funded studies**<br>Enter a statement with the following details:<br>• Initials of the authors who received each award<br>• Grant numbers awarded to each author<br>• The full name of each funder<br>• URL of each funder website<br>• Did the sponsors or funders play any role in the study design, data collection and analysis, decision to publish, or preparation of the manuscript?<br>• **NO** - Include this sentence at the end of your statement: *The funders had no role in study design, data collection and analysis, decision to publish, or preparation of the manuscript.*<br>• **YES** - Specify the role(s) played.<br><br>* typeset	
**Competing Interests**<br><br>Use the instructions below to enter a competing interest statement for this submission. On behalf of all authors, disclose any competing interests that could be perceived to bias this work—acknowledging all financial support and any other relevant financial or non-financial competing interests.<br><br>This statement is required for submission and **will appear in the published article** if the submission is accepted. Please make sure it is accurate and that any funding sources listed in your Funding Information later in the submission form are also declared in your Financial Disclosure statement.<br><br>View published research articles from	The authors have declared that no competing interests exist.

PLOS ONE for specific examples.<br><br>**NO authors have competing interests**<br><br>Enter: *The authors have declared that no competing interests exist.*<br><br>**Authors with competing interests**<br><br>Enter competing interest details beginning with this statement:<br><br>*I have read the journal's policy and the authors of this manuscript have the following competing interests: [insert competing interests here]*<br><br>* typeset	
**Ethics Statement**<br><br>Enter an ethics statement for this submission. This statement is required if the study involved:<br><br>• Human participants<br>• Human specimens or tissue<br>• Vertebrate animals or cephalopods<br>• Vertebrate embryos or tissues<br>• Field research<br><br>Write "N/A" if the submission does not require an ethics statement.<br><br>General guidance is provided below. Consult the submission guidelines for detailed instructions. **Make sure that all information entered here is included in the Methods section of the manuscript.**	N/A

**Format for specific study types**<br><br>**Human Subject Research (involving human participants and/or tissue)**<br>• Give the name of the institutional review board or ethics committee that approved the study<br>• Include the approval number and/or a statement indicating approval of this research<br>• Indicate the form of consent obtained (written/oral) or the reason that consent was not obtained (e.g. the data were analyzed anonymously)<br><br>**Animal Research (involving vertebrate animals, embryos or tissues)**<br>• Provide the name of the Institutional Animal Care and Use Committee (IACUC) or other relevant ethics board that reviewed the study protocol, and indicate whether they approved this research or granted a formal waiver of ethical approval<br>• Include an approval number if one was obtained<br>• If the study involved *non-human primates*, add *additional details* about animal welfare and steps taken to ameliorate suffering<br>• If anesthesia, euthanasia, or any kind of animal sacrifice is part of the study, include briefly which substances and/or methods were applied<br><br>**Field Research**<br><br>Include the following details if this study involves the collection of plant, animal, or other materials from a natural setting:<br>• Field permit number<br>• Name of the institution or relevant body that granted permission	
**Data Availability**<br><br>Authors are required to make all data underlying the findings described fully available, without restriction, and from the time of publication. PLOS allows rare exceptions to address legal and ethical concerns. See the PLOS Data Policy and FAQ for detailed information.	Yes - all data are fully available without restriction

A Data Availability Statement describing where the data can be found is required at submission. Your answers to this question constitute the Data Availability Statement and **will be published in the article**, if accepted.<br><br>**Important:** Stating 'data available on request from the author' is not sufficient. If your data are only available upon request, select 'No' for the first question and explain your exceptional situation in the text box.<br><br>Do the authors confirm that all data underlying the findings described in their manuscript are fully available without restriction?	
**Describe where the data may be found in full sentences. If you are copying our sample text, replace any instances of XXX with the appropriate details.**<br><br>• If the data are **held or will be held in a public repository**, include URLs, accession numbers or DOIs. If this information will only be available after acceptance, indicate this by ticking the box below. For example: *All XXX files are available from the XXX database (accession number(s) XXX, XXX.*).<br>• If the data are all contained **within the manuscript and/or Supporting Information files**, enter the following: *All relevant data are within the manuscript and its Supporting Information files.*<br>• If neither of these applies but you are able to provide **details of access elsewhere**, with or without limitations, please do so. For example:<br><br>*Data cannot be shared publicly because of [XXX]. Data are available from the XXX Institutional Data Access / Ethics Committee (contact via XXX) for researchers who meet the criteria for access to confidential data.*<br><br>*The data underlying the results presented in the study are available from (include the name of the third party*	All relevant data are within the manuscript and its Supporting Information files.

and contact information or URL).<br>• This text is appropriate if the data are owned by a third party and authors do not have permission to share the data.<br><br>* typeset	
Additional data availability information:	Tick here if the URLs/accession numbers/DOIs will be available only after acceptance of the manuscript for publication so that we can ensure their inclusion before publication.

Powered by Editorial Manager® and ProduXion Manager® from Aries Systems Corporation

Manuscript Click here to access/download;Manuscript;Manuscript.docx

Larvicidal properties of terpenoid-based nanoemulsions against the Dengue vector *Aedes aegypti* L. and their potential toxicity against non-target organism

Jonatas Lobato Duarte[1], Stéphane Duchon[2], Leonardo Delello Di Filippo[1], Marlus Chorilli[1], Vincent Corbel[2,3]

[1]Department of Drugs and Medicines, School of Pharmaceutical Sciences, São Paulo State University (UNESP), Araraquara, São Paulo, Brazil

[2] Institut de Recherche pour le Développement (IRD), MIVEGEC, Univ. Montpellier, CNRS, IRD, 911 Av Agropolis, 34 394 Montpellier, France

[3] Fundação Oswaldo Cruz (FIOCRUZ), Instituto Oswaldo Cruz (IOC), Laboratório de Fisiologia e Controle de Artrópodes Vetores (Laficave). Avenida Brasil, 4365 Manguinhos, Rio de Janeiro – RJ, CEP: 21040-360, Brazil

Abstract

The development of insecticide resistance in mosquitoes of public health importance has encouraged extensive research into innovative vector control methods. Terpenes are the largest among Plants Secondary Metabolites and have been increasingly studied for their potential as insecticidal control agents. Although promising, terpenes are insoluble in water, and they show low residual life which limits their application for vector control. In this study, we developed and evaluated the performances of terpenoid-based nanoemulsions (TNEs) containing myrcene and p-cymene against the dengue vector *Aedes aegypti* and investigated their potential toxicity against non-target organisms. Our results showed that myrcene and p-cymene showed moderate larvicidal activity against mosquito larvae compared to temephos an organophosphate widely used for mosquito control. However, we showed similar efficacy of TNEs against both susceptible and highly insecticide-resistant mosquitoes from French Guyana, hence suggesting an absence of cross-resistance with conventional insecticides. We also showed that TNEs remained effective for up to 45 days in laboratory conditions. The exposure of zebrafish to TNEs triggered behavioral changes in the fish at high doses but they did not alter the normal functioning of zebrafish organs, suggesting a good tolerability of non-target organisms to these molecules. Overall, this study provides new insights into the insecticidal properties and toxicity of terpenes and terpenoid-based formulations and confirms that TNE may offer interesting prospects for mosquito control as part of integrated vector management.

Key-words: dengue, *Aedes aegypti*, larval control, terpenes, p-cymene, myrcene, nanoemulsion

Introduction

Over the past decade, *Aedes Borne Diseases* (ABDs) such as dengue, zika, chikungunya, and yellow fever have become a global concern due to their emergence or re-emergence [1]. These diseases pose a significant threat to over half of the world's population, resulting in thousands of deaths annually, mainly in the Americas, South-East Asia, and Western Pacific regions [2]. Beyond their direct impact on human health and well-being, ABDs may cause tremendous economic impact [3,4], recently estimated to be US$ 150 billion over the period 1970-2017 [5].

Aedes aegypti L. is the primary dengue vector, with a widespread distribution worldwide, a high vectorial capacity, due to its close association with humans in urban settings [6]. *Ae. aegypti* distribution has increased recently due to global changes [7] and it may be more prevalent in temperate regions such as Europe due to the presence of suitable conditions for establishment [8]. The strategies for effectively controlling *Ae. aegypti* essentially rely on environmental management, community participation, and on the use of larviciding and spatial spraying (in case of outbreaks) [9]. A recent systematic review showed that mass trapping interventions, used in combination with classical vector control interventions, can be very efficient in reducing the density of *Ae. aegypti* and *Aedes albopictus* populations as well as viral transmission [10].

As far as chemical control is concerned, organic substances, such as pyrethroids, organophosphates, and to a lesser extent insect growth regulators are mainly used. However, the extensive and repeated use of those chemicals has caused the emergence and spread of insecticide resistant mosquitoes worldwide [11]. For example, recent studies have shown an increasing resistance of *Ae. aegypti* to pyrethroids [12–15] and organophosphates [16–18] due to the presence of target site modifications (eg kdr mutations for pyrethroids) and increased detoxification through the overexpression of P450, GST and esterase genes [19–21]. The occurrence of “super” insecticide-resistant *Ae. aegypti* mosquitoes have even been reported in Southeast Asia due to the presence of 3 different *Kdr* mutations in the same individuals [22]. This rapid and global increase in Aedes resistance makes it more difficult to control field populations with current public health pesticides and may potentially worsen the impact of ABDs worldwide. Moreover, chemicals can be toxic for non-target organisms and the environment [23,24], and as such, they are facing increasing regulatory constraints and citizen aversions.

Therefore, there`s an urgent need for more ecological, durable, and efficient approaches to control ABDs [25]. Natural products show interesting prospects in vector control because they are degraded more easily and are less harmful to the environment than conventional chemicals, and they may contribute to insecticide resistance management in mosquitoes [26]. Among natural products, essential oils (EO) have been extensively studied because they contain suitable active ingredients for the development of plant-based insecticides [27–30]. The monoterpenes represent about 90% of the EO composition and are the main ones responsible for larvicidal and repellent activities [31]. The *P-cymene* and myrcene are hydrocarbon monoterpenes that already showed high potential as larvicidal agents against *Ae. aegypti* [26,32–35]. However, due to the lipophilic characteristic of the terpenes and their high volatility, they may offer a limited efficacy against mosquitoes in a natural environment.

Consequently, nanoemulsions represent a promising tool for the encapsulation, protection, and release of lipophilic bioactive components such as terpens [36,37]. Nanoemulsions consist of two immiscible liquids and one or more stabilizers, usually surfactant agents, that may ensure better stability and efficacy of these molecules in water environment.

This study aimed to evaluate the effectiveness against insecticide-susceptible and highly resistant *Ae. aegypti* mosquitoes of terpene-based nanoemulsions (TNEs) containing *p*-cymene and myrcene. Biological assays were performed with successive concentrations of p-cymene and myrcene formulations, both alone and in combination, to determine concentration-response plots and estimate adequate metrics. The study also investigated any cross-resistance mechanism with conventional insecticides that may affect the effectiveness of TNEs. Finally, we assessed the acute toxicity of these nanoemulsions against non-target organisms, specifically Zebrafish.

Material and methods

Preparation of formulations

The TENs were obtained by a low-energy method as described in (DUARTE *et al submitted*). Briefly, an oil phase composed of the terpene (p-cymene or myrcene) (5% w/w) was mixed with the surfactants (Span® 80/Tween® 20) (5% w/w) (Table 1) under a magnetic stirrer and after homogenization, the aqueous phase (90% w/w) was added dropwise. The resulting nanoemulsions had a droplet size of approximately 120 nm and

a uniform distribution of particle size. They remained stable for up to 90 days, indicating good colloidal stability.

Table 1. Composition of the monoterpenes nanoemulsions.

	Monoterpene (%)	Tween 20 (%)	Span 80 (%)	Water
**P-cymene-NE**	5	4,7	3	90
**Myrcene-NE**	5	4,3	7	90

NE: Nanoemulsion

Biological material

Two different strains of *Ae. aegypti* were used in the study. The susceptible reference laboratory strain (Bora) originating from French Polynesia has no detectable resistance mechanism [38]. The strain is maintained at IRD for more than 20 years. The Guyana strain has been collected in French Guyana (Ile Royale) and shows high levels of resistance to pyrethroids [39] through the presence of V1016I and F1534C mutations at high frequencies and higher expression of multiple CYP450 genes [19]. The strain is also resistant to organophosphate through higher expression of Esterase genes [39].

Larvicidal activity and cross-resistance assessment

Larval bioassays were conducted according to the standardized protocol established by the World Health Organization [40]. The protocol involved exposing III-IV instar larvae to different concentrations of terpenes and terpene-based formulations and recording their mortality after 24 hours. The experiments were conducted under controlled conditions, where larvae of *Ae. aegypti* (Bora strain and Guyana strains) were kept at 25 ± 2 ºC, with a relative humidity of 75 ± 5%, and a light/dark cycle of 12 hours during the test. The experiments were carried out in triplicate, with four replicates of 25 larvae (n=100) per concentration, as well as a control. Temephos, an organophosphate insecticide commonly used for larval control, was used as the reference product.

Concentration-response curves were determined against both susceptible and pyrethroid resistance colonies of *Ae. aegypti* (Bora and Guyana) in order to assess potential cross-resistance. The resistance ratios (RR_{50}/RR_{95}) were determined by comparing the $LC_{50/95}$ obtained on the resistant strain and the susceptible ones. Evidence

for cross-resistance was demonstrated if resistance ratios ($RR_{50/95}$) exhibited confidence limits excluding the value of 1.

Interactions between monoterpenes

The existence of synergism between p-cymene and myrcene or Cym-NE and Myr-NE was also investigated by using the combination index (CI) described by Chou and Talalay (1984) [41], using the CalcuSyn software (Chou and Hayball 1996). This isobologram-based method is particularly well adapted to analyse multiple drug effects [42]. The combination index gives a quantitative measure of the interactions (synergism, antagonism, and summation) occurring between two insecticides. A CI = 1, <1, and >1 indicates an additive effect, a synergistic effect, and an antagonistic effect, respectively [43]

Residual activity of terpene-based formulations

For the residual activity, a stock solution of the TENs was prepared in the concentration of 40 mg/L and stored in a climatic chamber with controlled conditions (temperature: 27 °C and Relative humidity: 80%). Larval bioassays were carried out as previously described using a single dose of 40mg/L at different intervals of time (1, 7 15, 30, and 45 days) and mortality was recorded at 24H post-exposure.

Toxicity in a non-target organism

Animals

The animals used were adult zebrafish (*Danio rerio*) of the wild AB type, supplied by the company Piscicultura Power Fish, located in Itaguaí-RJ, Brazil. They were maintained in the Zebrafish Platform of the Drug Research Laboratory of the Federal University of Amapá, (UNIFAP- Brazil), with an adaptation period (40 days), a circadian rhythm of 12 hours, (light period from 7:00 a.m. to 7:00 p.m.), with controlled temperature (23 ± 2°C), and receiving commercial fish food (Alcon Colors, Santa Catarina, Brazil) twice a day [44]. The animal health and behaviour were monitored during all the analysis period (24 hours) and all researchers involved in the experiments have training in animal care. The experiments were authorized by the Ethics Committee on Animal Experimentation of the Federal University of Amapá (Brazil), registration number 006/2021.

Acute oral toxicity study

The acute toxicity evaluation of Cym-NE and Myr-NE in adult zebrafish was determined using the Limit Test as recommended by the Organization for Economic Cooperation and Development (OECD) 425 [45], with adaptations. The animals were separated into treatment groups (5 animals/group), kept fasting for 24 hours, weighed, and treated orally according to the methods described by [46]. Cym-NE was administered at doses of 2000 and 1750 mg/kg and Myr-NE at a dose of 2000 mg/kg, orally by gavage method, with the aid of a volumetric pipette (HTL Lab Solutions Co.), the volume was calculated according to the animal's weight [47].

Behavioural analysis and mortality

After the gavage procedure, the animals were observed for behavioral changes. The observed behavioral changes were classified as Stage I: increase in swimming activity; Spasms; tremors in the tail; Stage II: circular swimming; loss of posture; Stage III: clonus; loss of motility; animal still at the bottom of the aquarium and death [44]. In the absence of a response to mechanical stimulation and the absence of movement of the operculum, the animal was considered dead [44,47]. At the end of the observations (24 hours) the animals underwent euthanasia in anesthetic cooling, according to the American Guidelines of the Veterinary Medical Association for Animal Euthanasia [48].

Histopathological analysis

For histopathological analysis of the intestine, liver, and kidneys, the animals were fixed in Bouin solution for 24 hours and decalcified in EDTA solution (Tetraacetic Ethylenediamine Acid, Sigma Co., São Paulo, Brazil) for 48 hours. The samples were dehydrated in a series of alcohols (70, 80, 90, and 100%), diaphanized in xylol, and included in paraffin. The samples were sectioned in 5 μm using a microtome (Brand Rotary Microtome Cut 6062, Slee Medical, Germany), and histopathological analysis was performed after the tissue sections were cordoned with hematoxylin and eosin, as described by Souza et al., (2016). The images were analyzed under Olympus BX41-Micronal Microscope and photographed with MDCE-5C USB 2.0 (digital) camera.

Assessment of histopathological changes

The Index of Histopathological Changes (IHC) was calculated according to the levels of tissue alterations observed in the gills, liver, and kidneys. The changes were classified as levels I, II, and III, and the AHI value indicates whether the organ is healthy

(0 to 10), with changes from mild to moderate (11 to 20), with moderate to severe changes (21 and 50) or containing irreversible changes (> 100) [49,50]. Thus, the indexes were calculated according to the following equation:

$$I = \frac{\sum_{i-1}^{na} ai + 10 \sum_{i-1}^{nb} bi + 10^2 \sum_{i-1}^{nc} ci}{N}$$

Where: a: first stage changes; **b**: second stage changes; **c**: third stage changes; **na**: number of changes considered as the first stage; **nb**: number of changes considered as the second stage; **nc**: number of changes considered as the third stage; **N**: number of fishes analysed per treatment.

Statistical analysis

For larval bioassays, LC_{50} and LC_{90} values with their 95% confidence intervals were calculated from a log dose–probit mortality regression line using the SPSS software (IBM, USA). The results of acute oral toxicity obtained were expressed as the mean ± standard deviation (SD) of each experimental group. The ANOVA test was applied, followed by the Tukey test. The significance level considered was 5% ($p < 0.05$). The software used was the Prisma® Graph Pad (version 5.03).

Results

Larvicidal activity of terpenes and cross-resistance studies

Bioassay data for the susceptible mosquito strain are shown in Table 2 and Fig. 1. Temephos (reference product) showed high insecticidal activity against susceptible mosquito larvae, with a LC_{50} and LC_{99} of 0.001 and 0.00177 mg/L, respectively.

On the other hand, myrcene and p-cymene, showed moderate larvicidal activity against the susceptible BORA strain with LC_{50} values of 12.8 and 13.3 mg/L, respectively, and LC_{99} values of 29.5 and 47.9 mg/L, respectively.

The nanoemulsification process did not significantly affect the overall efficacy of the monoterpenes as LC_{50} and LC_{99} values of Myr-NE and Cym-NE did not differ than the one's of the free monoterpenes.

Figure 1. Concentration-response plots of terpenes against A*e. Aegypti* larvae: A – Bora strain with Cym and Cym-NE; B – Bora strain with Myr and Myr-NE; C – Guyana strain with Cym and Cym-Ne and D – Guyana strain with Myr and My-NE

Table 2. Larvicidal activity of monoterpenes, free and in nanoemulsions, against *Ae. aegypti* L. (Bora strain).

	n	X^2	df	*P value*	LC_{50} (95%CI)	LC_{99} (95%CI)
Temephos	3362	6.7	5	0.244	0.001 (0.00102-0.00108)	0.00177 (0.00168-0.00188)
Myrcene	3351	20	4	<0.001	12.8 (8.8-15.3)	29.5 (25.0-42.1)
Myr-NE	3352	44.7	5	<0.001	13.3 (7.1-17.8)	47.9 (36.9-79.9)
P-cymene	3366	188.3	5	0.003	16.0 (14.8-17.0)	29.4 (26.5-34.3)
Cym-NE	3356	9.00	4	0.061	14.0 (13.4-14.7)	24.7 (22.6-27.8)

Bioassays data for the resistant mosquito strain is shown in Table 3 and Figure 1. Our finding showed that the Guyana strain was highly resistant to temephos with RR_{50} and RR_{99} of 124 and 253, respectively. Our findings showed no cross-resistance of the Guyana strain to monoterpenes, with RR_{50} and RR_{95} ranging from 0.83 (95%CI 0.59 - 1.15) to 1.84 (1.34 - 2.52). Once again, no significant difference in efficacy was observed between free monoterpenes and nanoemulsions against the insecticide resistant *Ae. Aegypti* colony, suggesting that the nanoemulsification process does not impair the biological activity of the p-cymene and myrcene.

Table 3. Larvicidal activity of monoterpenes, free and in nanoemulsions against insecticide-resistant *Ae. aegypti* (Guyana strain).

	N	X^2	df	P value	LC_{50} (95% CI)	LC_{99} (95% CI)	RR_{50} (95% CI)	RR_{95} (95% CI)
Temephos	2250	30.8	5	<0.001	0.150 (0.05-0.19)	0.450 (0.336-1.743)	124.44 (103.40-149.77)	253.03 (160.90 - 397.93)
Myrcene	2000	7.8	4	0.099	17.3 (15.0-19.0)	37.8 (33.4-46.2)	1.36 (1.22 - 1.53)	1.30 (1.035 - 1.64)
Myr-NE	2000	12.2	4	0.016	17.3 (14.9-19.0)	34.6 (30.3 -43.8)	1.25 (1.07 - 1.47)	0.83 (0.59 - 1.15)
P-cymene	2000	14.5	4	0.006	21.8 (18.8-23.9)	41.8 (35.7-58.9)	1.24 (1.07 - 1.54)	1.55 (1.05 - 2.29)
Cym-NE	2000	14.9	4	0.005	21.0 (18.0-23.0)	42.1 (35.8-58.3)	1.39 (1.20 - 1.60)	1.84 (1.34 - 2.52)

Interactions between monoterpenes

Here, a 1:1 ratio (myrcene:p-cymene) was adopted for the combination considering that both terpenes had similar LC_{50} against both susceptible and resistant mosquitoes. Concentration-response plots of terpenes, alone and in a mixture, are shown in Fig. 2.

Figure 2 – Concentrations effect curves of the combination of A - Myrcene:p-cymene (1:1) and B -Myr-NE:Cym-NE (1:1) and Interaction curves between C - Myrcene:p-cymene (1:1) and D -Myr-NE:Cym-NE (1:1)

Our findings showed that the combination of terpenes did not kill a higher proportion of mosquitoes than the sum of the 2 terpenes taken separately. Combination indexes (CI) ranged from 1 to 1.5 hence indicating additive and slight antagonism at high concentrations, respectively according to the classification of CHOU & TALALAY, [41] (Figure 2 C and D).

Residual activity

The results showed that the TNEs remain effective (providing 100% mortality) up to 45 days of storage (Fig. 3), under laboratory-controlled conditions of temperature (27 °C) and relative humidity (80%).

Figure 3. Residual activity of Cym-NE and Myr-NE against susceptible larvae of *Ae. Aegypti*. numbers in the bars represent the sample size tested.

Toxicity of terpenes against non-target organism

The treatment of zebrafish with Cym-NE (1750 and 2000 mg/kg) and Myr-NE (2000 mg/kg) triggered behavioral changes in zebrafish as shown in Table 4. However, mortality was observed in the group of animals treated at the dose of 2000 mg/kg of Ne-Cym. The signs of stress were an increase in swimming activity, tremors in the tail, loss of posture and motility, circular swimming, permanence at the bottom of the aquarium, and death.

Table 4 - Effect of oral treatment with Ne-Cym and Ne-Myr and control (water) over zebrafish behavior and mortality.

Group	Doses	Stage I	Stage II	Stage III	Total	%
**Control (water)**	2µl	1/3	0/2	0/4	1/9	11.1
**Cym-NE**	1750 mg/kg	2/3	2/2	2/4	6/9	66.6
	2000 mg/kg	1/3	2/2	1/4	4/9	44.4
**Myr-NE**	500 mg/kg	2/3	2/2	2/4	6/9	66.6

The main alterations observed in the tissues were: dilation of presents vessels in villi and hypertrophy of caliciform cells in the intestine, cytoplasmic vacuolization and nuclear atypia in the hepatocytes, prevalence of tubular alterations, such as mild tubular hyaline degeneration in the kidneys (Fig. 5). The IHC calculated was less than 10, which classifies these organs as normal, because the alterations recorded were not able to alter the normal functioning of the organs.

Figure 4. Histopathological changes observed in the intestine (A and B), liver (Cand D) and kidneys (E and F). In A and B intestinal tissue with caliciform cells (GC), villi (V), Muscle layer (MC), villi degeneration (VD) and caliciform cell hyperplasia (HpGC); In C and D normal hepatocytes (H), Cytoplasmatic vacuolization (CV), nuclear atrophy (NA); In E and F tubules (Tb), Lymphoid tissue (TL), Increase in tubular lumen (ALT), mild tubular hyaline degeneration (DHT). H&E staining, IHC in the intestine (G), liver (H) and kidneys (I) of adult zebrafish in the acute oral toxicity test performed using Ne-Cym (1750 e 2000 mg/kg) e Ne-Myr (2000 mg/kg). Data show the mean ± SD (n = 5/group). Statistical analysis was performed through one-way ANOVA followed by the post hoc Tukey test.

Discussion

In this study, we investigated the larvicidal activity of myrcene and *p*-cymene against the dengue vector *Ae. aegypti* and their potential toxicity against non-target organisms.

Our findings showed that myrcene and p-cymene exhibited moderate insecticidal activity against *Ae. aegypti* larvae when compared to the reference product Temephos. The lower efficacy of terpenes against mosquitoes has been reported previously [51,52].

For example, Lee and Ahn (2013) reported that myrcene had a LC_{50} of 36 mg/L, against third-instar larvae which is even higher than the LC_{50} reported in the present study [51]. In contrast, Lucia et al. (2017) reported a lower LC_{50} (12.49 mg/L) for p-cymene against third instar larvae [53]. These findings suggest that the potency of these compounds as mosquito control agents varies according to the studies and several factors such as the source and composition of the monoterpenes, experimental conditions, and mosquito species probably impact on the outcomes. Although TNEs show moderate larvicidal activity, our findings showed an absence of cross-resistance with commonly used pesticides and this offers interesting prospects for vector control considering that mosquito resistance is becoming an increasing threat to the prevention and control of mosquito borne diseases (Corbel et al 2016, 2018). Indeed, there’s very limited number of insecticides having a different mode of action than the big “four” classes (i.e. pyrethroids, carbamates, organophosphate, organochlorines) and TNEs could then enrich the panel of vector control products used as part of integrated vector management.

Our study also compared the efficacy of free terpenes *versus* terpene-based nanoemulsions against *Ae. aegypti* mosquitoes. Our findings showed that nanoemulsions did not increase the efficacy of both myrcene and p-cymene against mosquito larvae. These results contrast with other findings showing an enhanced insecticidal activity of nanoemulsified terpenes or essential oils compared to non-encapsulated components [54,55]. For example, the studies by Almadiy and Nenaah (2022) and Ferreira et al. (2020) used different essential oils and different particle sizes for their nanoemulsions. Alamdy used *Origanum vulgare* essential oil while Ferreira et al. (2020) used the Essential Oil of *Siparuna guianensis*. Additionally, the particle size of the nanoemulsions in Alamdy's study was 60 nm while Ferreira et al. reported a particle size of around 160 nm. These differences in the chemical composition of the essential oils and the physicochemical characteristics of the nanoemulsions can affect the interaction of the terpenes with mosquito larvae and explain the different outcomes observed in our study compared to previous one’s.

Despite that, our study showed a residual efficacy of the nanoemulsions for at least 45 days in a climatic chamber at constant temperature and humidity. This suggests that the use of nanoemulsions may prevent the volatilization of terpenoids and provide long-lasting activity as previously reported ([56]. However, one bias of our study is that we couldn’t compare the residual activity of the free terpenes *versus* encapsulated terpenes, due to a shortage of mosquitoes. Morover, it is important to highlight that

laboratory conditions do not always reflect the field situation as external factors such as sunlight, rain, pollutants, etc can strongly reduce the residual efficacy of larvicides [57]. Further investigations are needed to assess the longevity of terpenes and TNEs in both laboratory and simulated field conditions.

Our research also investigated whether a combination of p-cymene and myrcene may be more effective at killing mosquitoes than each molecule used alone. The interest in using a combination of insecticides results in the fact that it may be possible to achieve better control of a mosquito population while reducing the doses of each compound, hence making this an environmentally friendly and cost-effective solution [58]. Our findings however showed that the combination of terpenes did not kill a higher proportion of mosquitoes than the sum of the 2 terpenes taken separately, indicating additive or even slight antagonism at high concentrations. These findings contrast with other studies that have reported additive to synergistic interactions between various monoterpenes (e.g. terpinene, limonene, carvacrol, anethole, peroxide ascaridole, p-cymene and methyl isoeugenol) against mosquito larvae [59,60]. It appears that the methodology used to assess the synergy and the compositions of terpenoid combinations differ between studies and results cannot be directly compared. In our study, the lack of synergistic interactions between p-cymene and myrcene might be explained by the fact that both terpenes act on the same target sites (i.e. mutually exclusive inhibitors) hence causing competitive inhibition for the same receptor, especially when concentrations increased (saturation of the system). More work is needed to better understand the interactions and mode of action of terpenoids in insects in order to select best active ingredients in combination.

Finally, we investigated the potential toxicity of the p-cymene and myrcene nanoemulsions against non-target organisms (fish) considering that these molecules may be potentially deployed in mosquito breeding habitats. Our results showed that TNEs lead to behavioral and histological changes in the Zebrafish at high concentrations. These alterations may lead to changes in body weight, food consumption, behavior, blood circulation, and reproductive functions [61]. Everds et al. (2013) state however that in toxicity trials, it is common to observe signs of stress in animals [62] and these histological alterations are considered normal, and not able to alter the normal functioning of the fish organs [63,64]. Although it is unlikely that p-cymene and myrcene pose a significant hazard to non-target organisms, there is a need to assess their potential toxicity in simulated field conditions using the doses that may be applied in the field.

Conclusions

Overall, our findings showed that TNEs have potential for vector control particularly as part of integrated vector management with the scope to preserve the susceptibility of mosquito populations to public health insecticides. Our findings suggest that the incorporation of monoterpenes into nanoemulsions is a promising approach to improve the solubility of these substances in aqueous media without organic solvents, which reinforces the vector control potential of these molecules.

Acknowledgments

We thank all team from IRD-MIVEGEC for their support during the biological tests.

Funding

This project has received funding from Sao Paulo research foundation-FAPESP under the grants n°2021/11487-4 and 2019/25125-7. This project also receives a co-funding from the European Union HORIZON-MSCA-2021-SE-01 (INOVEC project), under the grant n°101086257. Views and opinions expressed are however those of the author(s) only and do not necessarily reflect those of the European Union or the European Research Executive Agency (REA). Neither the European Union nor the REA can be held responsible for them.

References

1. Wilder-Smith A, Ooi EE, Horstick O, Wills B. Dengue. The Lancet. 2019;393: 350–363. doi:10.1016/S0140-6736(18)32560-1

2. Bhatt S, Gething PW, Brady OJ, Messina JP, Farlow AW, Moyes CL, et al. The global distribution and burden of dengue. Nature 2013 496:7446. 2013;496: 504–507. doi:10.1038/nature12060

3. Bradshaw CJA, Leroy B, Bellard C, Roiz D, Albert C, Fournier A, et al. Massive yet grossly underestimated global costs of invasive insects. Nat Commun. 2016;7: 12986. doi:10.1038/ncomms12986

4. Romero IC. A gestão de doenças infecto-contagiosas: o impacto do dengue na saúde pública. Trabalho de Conclusão de curso, Universidade Estadual Paulista Júlio de Mesquita FIlho. 2014.

5. Diagne C, Leroy B, Vaissière AC, Gozlan RE, Roiz D, Jarić I, et al. High and rising economic costs of biological invasions worldwide. Nature 2021 592:7855. 2021;592: 571–576. doi:10.1038/s41586-021-03405-6

6. Higa Y. Dengue Vectors and their Spatial Distribution. Trop Med Health. 2011;39: S17–S27. doi:10.2149/tmh.2011-S04

7. Kraemer MUG, Sinka ME, Duda KA, Mylne AQN, Shearer FM, Barker CM, et al. The global distribution of the arbovirus vectors Aedes aegypti and Ae. albopictus. Elife. 2015;4. doi:10.7554/ELIFE.08347

8. Corbel V, Durot C, Achee NL, Chandre F, Coulibaly MB, David J-P, et al. Second WIN International Conference on “Integrated approaches and innovative tools for combating insecticide resistance in vectors of arboviruses”, October 2018, Singapore. Parasit Vectors. 2019;12: 331. doi:10.1186/s13071-019-3591-8

9. Roiz D, Wilson AL, Scott TW, Fonseca DM, Jourdain F, Müller P, et al. Integrated Aedes management for the control of Aedes-borne diseases. PLoS Negl Trop Dis. 2018;12: e0006845. doi:10.1371/JOURNAL.PNTD.0006845

10. Jaffal A, Fite JI, Baldet TI, Delaunay P, dé ric Jourdain F, Mora-Castillo R, et al. Current evidences of the efficacy of mosquito mass-trapping interventions to reduce Aedes aegypti and Aedes albopictus populations and Aedes-borne virus transmission. PLoS Negl Trop Dis. 2023;17: e0011153. doi:10.1371/JOURNAL.PNTD.0011153

11. Moyes CL, Vontas J, Martins AJ, Ng LC, Koou SY, Dusfour I, et al. Contemporary status of insecticide resistance in the major Aedes vectors of arboviruses infecting humans. PLoS Negl Trop Dis. 2017;11. doi:10.1371/JOURNAL.PNTD.0005625

12. Melo Costa M, Campos KB, Brito LP, Roux E, Melo Rodovalho C, Bellinato DF, et al. Kdr genotyping in Aedes aegypti from Brazil on a nation-wide scale from 2017 to 2018. Sci Rep. 2020;10. doi:10.1038/S41598-020-70029-7

13. Rahman RU, Cosme LV, Costa MM, Carrara L, Lima JBP, Martins AJ. Insecticide resistance and genetic structure of Aedes aegypti populations from Rio de Janeiro State, Brazil. Benoit JB, editor. PLoS Negl Trop Dis. 2021;15: e0008492. doi:10.1371/journal.pntd.0008492

14. Smith LB, Kasai S, Scott JG. Pyrethroid resistance in Aedes aegypti and Aedes albopictus: Important mosquito vectors of human diseases. Pestic Biochem Physiol. 2016;133: 1–12. doi:10.1016/j.pestbp.2016.03.005

15. Amelia-Yap ZH, Chen CD, Sofian-Azirun M, Low VL. Pyrethroid resistance in the dengue vector Aedes aegypti in Southeast Asia: present situation and prospects for management. Parasit Vectors. 2018;11: 332. doi:10.1186/s13071-018-2899-0

16. Campos KB, Martins AJ, Rodovalho C de M, Bellinato DF, Dias L dos S, Macoris M de L da G, et al. Assessment of the susceptibility status of Aedes aegypti (Diptera: Culicidae) populations to pyriproxyfen and malathion in a nation-wide monitoring of insecticide resistance performed in Brazil from 2017 to 2018. Parasit Vectors. 2020;13. doi:10.1186/S13071-020-04406-6

17. Badolo A, Sombié A, Pignatelli PM, Sanon A, Yaméogo F, Wangrawa DW, et al. Insecticide resistance levels and mechanisms in Aedes aegypti populations in and around Ouagadougou, Burkina Faso. PLoS Negl Trop Dis. 2019;13: e0007439. doi:10.1371/journal.pntd.0007439

18. Rodríguez MM, Bisset J, de Fernandez DM, Lauzán L, Soca A. Detection of Insecticide Resistance in *Aedes aegypti* (Diptera: Culicidae) from Cuba and Venezuela. J Med Entomol. 2001;38: 623–628. doi:10.1603/0022-2585-38.5.623

19. Faucon F, Dusfour I, Gaude T, Navratil V, Boyer F, Chandre F, et al. Identifying genomic changes associated with insecticide resistance in the dengue mosquito *Aedes aegypti* by deep targeted sequencing. Genome Res. 2015;25: 1347–1359. doi:10.1101/gr.189225.115

20. Cattel J, Minier M, Habchi- Hanriot N, Pol M, Faucon F, Gaude T, et al. Impact of selection regime and introgression on deltamethrin resistance in the arbovirus vector *Aedes aegypti* – a comparative study between contrasted situations in <scp>New Caledonia</scp> and <scp>French Guiana</scp>. Pest Manag Sci. 2021;77: 5589–5598. doi:10.1002/ps.6602

21. Cattel J, Faucon F, Le Péron B, Sherpa S, Monchal M, Grillet L, et al. Combining genetic crosses and pool targeted DNA-seq for untangling genomic variations associated with resistance to multiple insecticides in the mosquito Aedes aegypti. Evol Appl. 2019;13: 303–317. doi:10.1111/EVA.12867

22. Kasai S, Itokawa K, Uemura N, Takaoka A, Furutani S, Maekawa Y, et al. Discovery of super–insecticide-resistant dengue mosquitoes in Asia: Threats of concomitant knockdown resistance mutations. Sci Adv. 2022;8. doi:10.1126/SCIADV.ABQ7345/SUPPL_FILE/SCIADV.ABQ7345_SM.PDF

23. Vieira Santos VS, Caixeta ES, Campos Júnior EO de, Pereira BB. Ecotoxicological effects of larvicide used in the control of *Aedes aegypti* on nontarget organisms: Redefining the use of pyriproxyfen. J Toxicol Environ Health A. 2017;80: 155–160. doi:10.1080/15287394.2016.1266721

24. Abe FR, Coleone AC, Machado AA, Gonçalves Machado-Neto J. Ecotoxicity and Environmental Risk Assessment of Larvicides Used in the Control of *Aedes aegypti* to *Daphnia magna* (Crustacea, Cladocera). J Toxicol Environ Health A. 2014;77: 37–45. doi:10.1080/15287394.2014.865581

25. Sugumar S, Clarke SK, Nirmala MJ, Tyagi BK, Mukherjee A, Chandrasekaran N. Nanoemulsion of eucalyptus oil and its larvicidal activity against Culex quinquefasciatus. Bull Entomol Res. 2014;104: 393–402. doi:10.1017/S0007485313000710

26. Silva IMA, Martins GF, Melo CR, Santana AS, Faro RRN, Blank AF, et al. Alternative control of Aedes aegypti resistant to pyrethroids: lethal and sublethal effects of monoterpene bioinsecticides. Pest Manag Sci. 2018;74: 1001–1012. doi:10.1002/ps.4801

27. Duarte JL, Maciel de Faria Motta Oliveira AE, Pinto MC, Chorilli M. Botanical insecticide–based nanosystems for the control of Aedes (Stegomyia) aegypti larvae. Environmental Science and Pollution Research. 2020;27: 28737–28748. doi:10.1007/s11356-020-09278-y

28. Duarte JL, Amado JRR, Oliveira AEMFM, Cruz RAS, Ferreira AM, Souto RNP, et al. Evaluation of larvicidal activity of a nanoemulsion of Rosmarinus officinalis essential oil. Revista Brasileira de Farmacognosia. 2015;25: 189–192. doi:j.bjp.2015.02.010

29. Rawani A, Ghosh A, Chandra G. Mosquito larvicidal and antimicrobial activity of synthesized nano-crystalline silver particles using leaves and green berry extract of Solanum nigrum L. (Solanaceae: Solanales). Acta Trop. 2013;128: 613–622. doi:10.1016/j.actatropica.2013.09.007

30. Oliveira AEMFM, Duarte JL, Cruz RAS, Souto RNP, Ferreira RMA, Peniche T, et al. Pterodon emarginatus oleoresin-based nanoemulsion as a promising tool for Culex quinquefasciatus (Diptera: Culicidae) control. J Nanobiotechnology. 2017;15. doi:10.1186/s12951-016-0234-5

31. Pavela R. Essential oils for the development of eco-friendly mosquito larvicides: A review. Ind Crops Prod. 2015;76: 174–187. doi:10.1016/j.indcrop.2015.06.050

32. Cheng SS, Lin CY, Chung MJ, Liu YH, Huang CG, Chang ST. Larvicidal activities of wood and leaf essential oils and ethanolic extracts from Cunninghamia konishii Hayata against the dengue mosquitoes. Ind Crops Prod. 2013;47: 310–315. doi:10.1016/j.indcrop.2013.03.016

33. Cheng S-S, Chang H-T, Lin C-Y, Chen P-S, Huang C-G, Chen W-J, et al. Insecticidal activities of leaf and twig essential oils from *Clausena excavata* against *Aedes aegypti* and *Aedes albopictus* larvae. Pest Manag Sci. 2009;65: 339–343. doi:10.1002/ps.1693

34. Cheng SS, Chua MT, Chang EH, Huang CG, Chen WJ, Chang ST. Variations in insecticidal activity and chemical compositions of leaf essential oils from Cryptomeria japonica at different ages. Bioresour Technol. 2009;100: 465–470. doi:10.1016/J.BIORTECH.2007.11.060

35. Ríos N, Stashenko EE, Duque JE. Entomologia A Journal on Insect Diversity and Evolution Evaluation of the insecticidal activity of essential oils and their mixtures against Aedes aegypti (Diptera: Culicidae). Rev Bras Entomol. 2017;61: 307–311. doi:10.1016/j.rbe.2017.08.005

36. McClements DJ. Nanoemulsions versus microemulsions: terminology, differences, and similarities. Soft Matter. 2012;8: 1719–1729. doi:10.1039/C2SM06903B

37. McClements DJ. Edible nanoemulsions: fabrication, properties, and functional performance. Soft Matter. 2011;7: 2297. doi:10.1039/c0sm00549e

38. Marcombe S, Poupardin R, Darriet F, Reynaud S, Bonnet J, Strode C, et al. Exploring the molecular basis of insecticide resistance in the dengue vector Aedes aegypti: A case study in Martinique Island (French West Indies). BMC Genomics. 2009;10: 494. doi:10.1186/1471-2164-10-494

39. Epelboin Y, Wang L, Giai Gianetto Q, Choumet V, Gaborit P, Issaly J, et al. CYP450 core involvement in multiple resistance strains of Aedes aegypti from French Guiana highlighted by proteomics, molecular and biochemical studies. PLoS One. 2021;16: e0243992. doi:10.1371/journal.pone.0243992

40. WORLD HEALTH ORGANIZATION. Guidelines for laboratory and field testing of mosquito larvicides. World Health Organization. 2005; 1–41. doi:Ref: WHO/CDS/WHOPES/GCDPP/2005.11

41. Chou T-C, Talalay P. Analysis of combined drug effects: a new look at a very old problem. Trends Pharmacol Sci. 1983;4: 450–454. doi:10.1016/0165-6147(83)90490-X

42. Tallarida RJ. The interaction index: a measure of drug synergism. Pain. 2002;98: 163–168. doi:10.1016/S0304-3959(02)00041-6

43. Darriet F, Corbel V. Laboratory evaluation of pyriproxyfen and spinosad, alone and in combination, against Aedes aegypti larvae. J Med Entomol. 2006;43: 1190–1194. doi:10.1603/0022-2585(2006)43[1190:LEOPAS]2.0.CO;2

44. Sousa GC de, Duarte JLD, Fernandes CP, Moyado AV, Navarrete A, Carvalho Jo carlos T. Obtainment and Study of the Toxicity of Perillyl Alcohol Nanoemulsion on Zebrafish (Danio rerio). J Nanomed Res. 2016;4: 18–20. doi:10.15406/jnmr.2016.04.00093

45. OECD. Test No. 425: Acute Oral Toxicity: Up-and-Down Procedure. OECD; 2022. doi:10.1787/9789264071049-en

46. Borges RS, Keita H, Ortiz BLS, dos Santos Sampaio TI, Ferreira IM, Lima ES, et al. Anti-inflammatory activity of nanoemulsions of essential oil from Rosmarinus officinalis L.: in vitro and in zebrafish studies. Inflammopharmacology. 2018;26: 1057–1080. doi:10.1007/S10787-017-0438-9

47. de Souza GC, Viana MD, Goés LDM, Sanchez-Ortiz BL, Silva GAD, Pinheiro W de S, et al. Reproductive toxicity of the hydroethanolic extract of the flowers of Acmella oleracea and spilanthol in zebrafish: In vivo and in silico evaluation. Hum Exp Toxicol. 2020;39: 127–146. doi:10.1177/0960327119878257

48. American Veterinary Medical Association (AVMA). Guidelines for the euthanasia of animals. 2020th ed. Schaumburg; 2020.

49. Carvalho JCT, Keita H, Santana GR, de Souza GC, dos Santos IVF, Amado JRR, et al. Effects of Bothrops alternatus venom in zebrafish: a histopathological study. Inflammopharmacology. 2018;26: 273–284. doi:10.1007/S10787-017-0362-Z

50. Poleksic V, Mitrovic-Tutundzic V. Fish gills as a monitor of sublethal and chronic effects of pollution. In: Muller R, Lloyd R, editors. In Sublethal and Chronic Efects of Pollutants on Freshwater Fish. Farnhan:Oxford: Fishing New Books ltd; 1994. pp. 339–352.

51. Lee DC, Ahn YJ. Laboratory and Simulated Field Bioassays to Evaluate Larvicidal Activity of Pinus densiflora Hydrodistillate, Its Constituents and Structurally Related Compounds against Aedes albopictus, Aedes aegypti and Culex pipiens pallens in

Relation to Their Inhibitory Effects on Acetylcholinesterase Activity. Insects. 2013;4: 217–229. doi:10.3390/INSECTS4020217

52. Santos SRL, Silva VB, Melo MA, Barbosa JDF, Santos RLC, de Sousa DP, et al. Toxic Effects on and Structure-Toxicity Relationships of Phenylpropanoids, Terpenes, and Related Compounds in Aedes aegypti Larvae. Vector-Borne and Zoonotic Diseases. 2010;10: 1049–1054. doi:10.1089/vbz.2009.0158

53. Lucia A, Zerba E, Masuh H. Knockdown and larvicidal activity of six monoterpenes against Aedes aegypti (Diptera: Culicidae) and their structure-activity relationships. Parasitol Res. 2013;112: 4267–4272. doi:10.1007/s00436-013-3618-6

54. Ferreira RM dos A, D'haveloose NP, Cruz RAS, Araújo RS, Carvalho JCT, Rocha L, et al. Nano-emulsification Enhances the Larvicidal Potential of the Essential Oil of Siparuna guianensis (Laurales: Siparunaceae) Against Aedes (Stegomyia) aegypti (Diptera: Culicidae). J Med Entomol. 2020;57: 788–796. doi:10.1093/jme/tjz221

55. Almadiy AA, Nenaah GE. Essential oil of *Origanum vulgare* , its nanoemulsion and bioactive monoterpenes as eco-friendly novel green pesticides for controlling *Aedes aegypti* , the common vector of Dengue virus. Journal of Essential Oil Research. 2022;34: 424–438. doi:10.1080/10412905.2022.2091673

56. Mustafa IF, Hussein MZ. Synthesis and Technology of Nanoemulsion-Based Pesticide Formulation. Nanomaterials 2020, Vol 10, Page 1608. 2020;10: 1608. doi:10.3390/NANO10081608

57. Marcombe S, Darriet F, Agnew P, Etienne M, Yp-Tcha MM, Yébakima A, et al. Field Efficacy of New Larvicide Products for Control of Multi-Resistant Aedes aegypti Populations in Martinique (French West Indies). Am J Trop Med Hyg. 2011;84: 118–126. doi:10.4269/AJTMH.2011.10-0335

58. Darriet F, Corbel V. Laboratory Evaluation of Pyriproxyfen and Spinosad, Alone and in Combination, Against Aedes aegypti Larvae. J Med Entomol. 2006;43: 1190–1194. doi:10.1093/JMEDENT/43.6.1190

59. Pavela R, Maggi F, Kulheim C, Urzua A, Palacios SM, Scalerandi E, et al. Understanding Synergistic Toxicity of Terpenes as Insecticides: Contribution of Metabolic Detoxification in Musca domestica. 2018 [cited 12 Mar 2023]. doi:10.3389/fpls.2018.01579

60. Dhinakaran SR, Mathew N, Munusamy S. Synergistic terpene combinations as larvicides against the dengue vector Aedes aegypti Linn. Drug Dev Res. 2019;80: 791–799. doi:10.1002/DDR.21560

61. Carvalho JCT, Souza GC De, Silva IDR Da, Viana MD, Melo NC De, Sánchez-Ortiz BL, et al. Acute Toxicity of the Hydroethanolic Extract of the Flowers of Acmella oleracea L. in Zebrafish (Danio rerio): Behavioral and Histopathological Studies. Pharmaceuticals (Basel). 2019;12. doi:10.3390/PH12040173

62. Everds NE, Snyder PW, Bailey KL, Bolon B, Creasy DM, Foley GL, et al. Interpreting Stress Responses during Routine Toxicity Studies. Toxicol Pathol. 2013;41: 560–614. doi:10.1177/0192623312466452

63. de Souza AA, Ortíz BLS, Borges SF, Pinto AVP, Ramos R da S, Pena IC, et al. Acute Toxicity and Anti-Inflammatory Activity of Trattinnickia rhoifolia Willd (Sucuruba)

Using the Zebrafish Model. Molecules 2022, Vol 27, Page 7741. 2022;27: 7741. doi:10.3390/MOLECULES27227741

64. Holanda FH, Ribeiro AN, Sánchez-Ortiz BL, de Souza GC, Borges SF, Ferreira AM, et al. Anti-inflammatory potential of baicalein combined with silk fibroin protein in a zebrafish model (Danio rerio). Biotechnology Letters 2022 45:2. 2022;45: 235–253. doi:10.1007/S10529-022-03334-Y

Figure 1

Click here to access/download;Figure;Figure 1.tiff

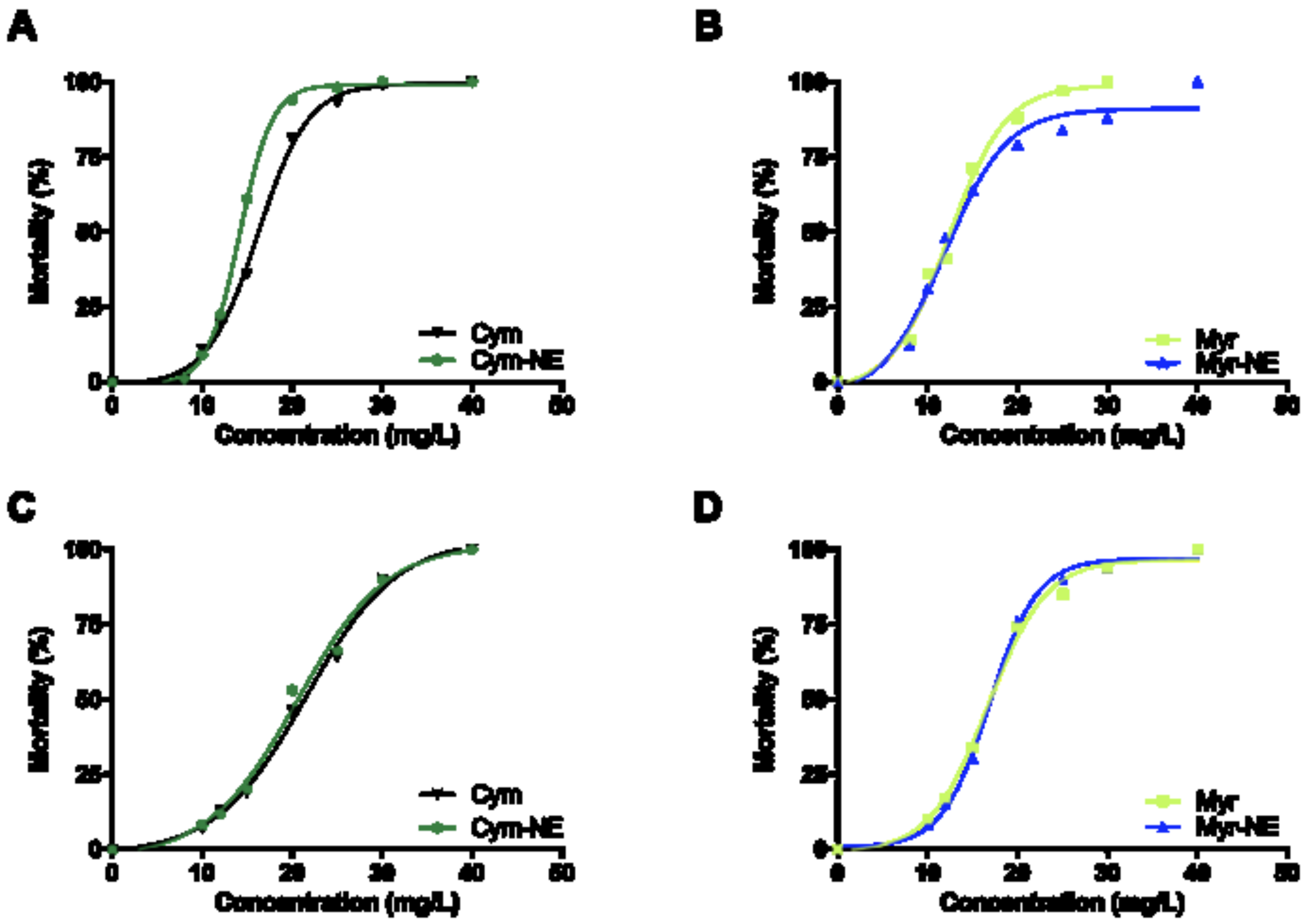

 Click here to access/download;Figure;Figure 2.tiff

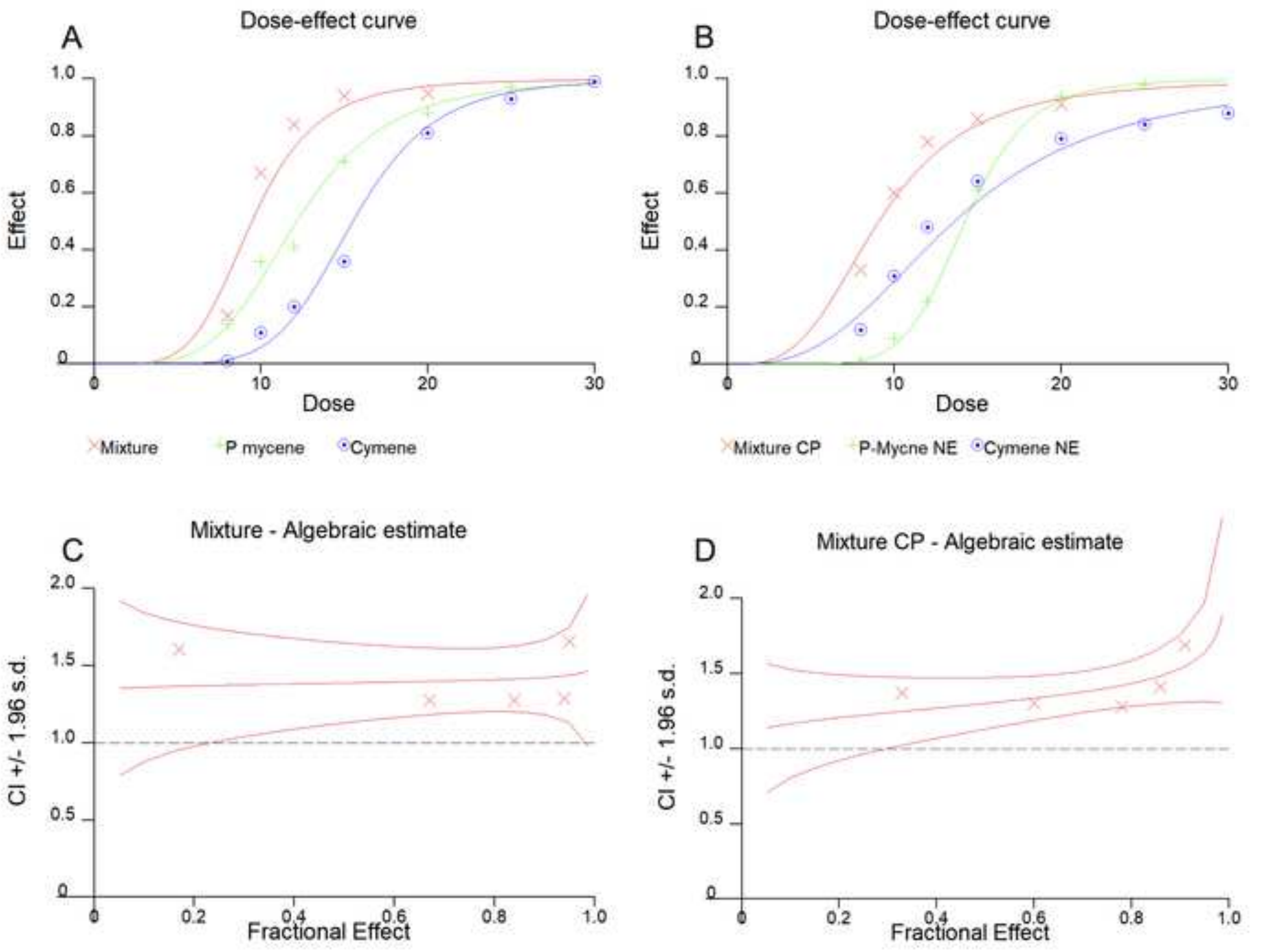

Figure3

Click here to access/download;Figure;Figure 3.tiff

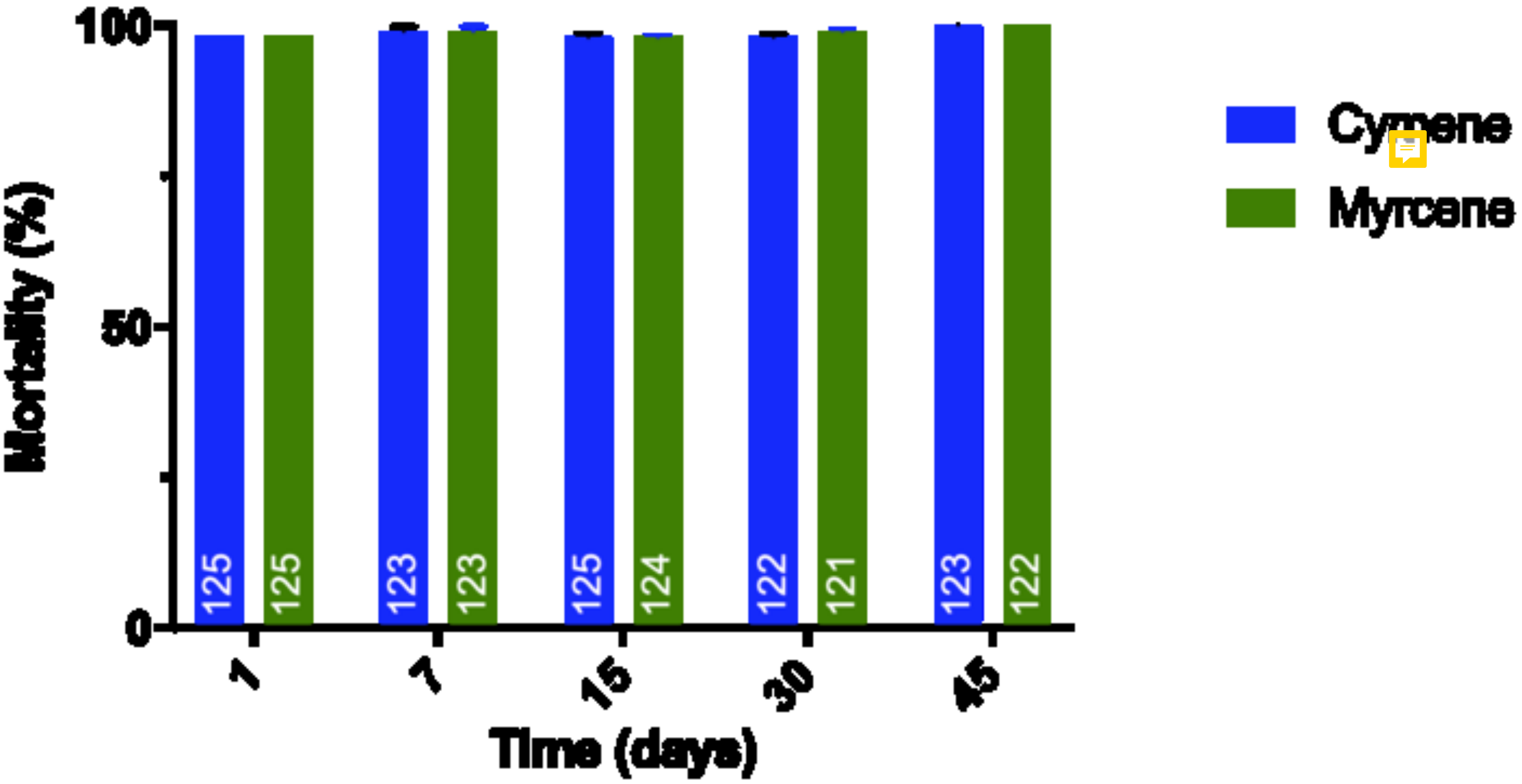

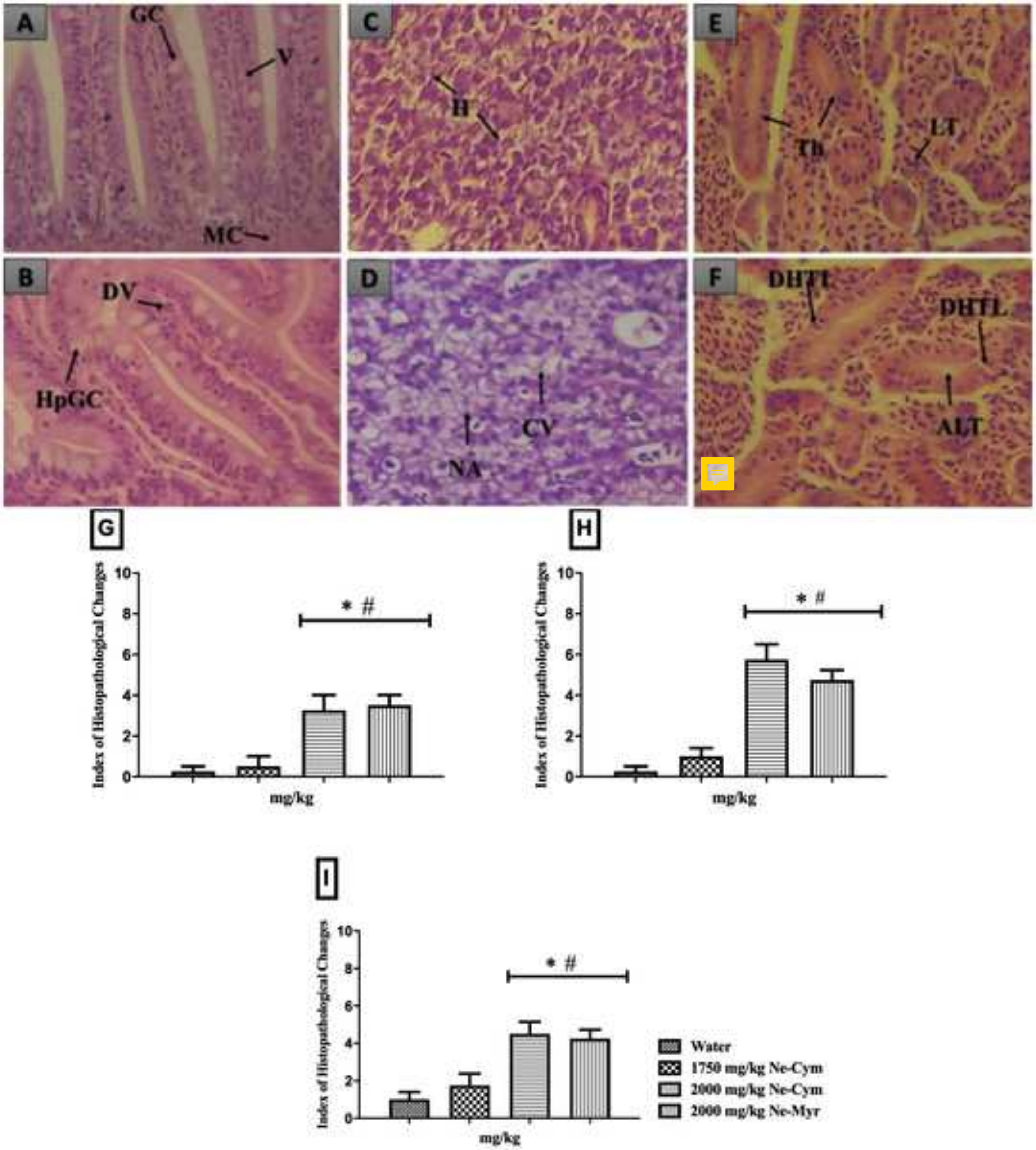
A
GC
V
MC
B
DV
HpGC
C
H
D
CV
NA
E
Tb
LT
F
DHTL
DHTL
ALT
G
H
I
Index of Histopathological Changes
10
8
6
4
2
0
* #
mg/kg
Water
1750 mg/kg Ne-Cym
2000 mg/kg Ne-Cym
2000 mg/kg Ne-Myr
