# Supplementary material for: Larvicidal properties of terpenoid-based nanoemulsions against the dengue vector Aedes aegypti L. and their potential toxicity against non-target organism
Source: PLoS One. 2024 Feb 7;19(2):e0293124. doi: 10.1371/journal.pone.0293124 (PMC10849395; doi:10.1371/journal.pone.0293124)
Supplement: Attachment — Submitted filename: Comments Reviewer.docx [file pone.0293124.s001.docx]

Introduction

Good explanations on the introduction about the mechanisms of resistant and objective of this study. Very clear and on the points for a research statements. The experiment design is much targeted and design well. Appropriate in this context.

1. Line 56-65 can be mentioned more comprehensive, especially the alternative method. You are not using those method and not relevant in your study scope
2. Explain more on the Nano emulsions technology for slow release. How the mechanisms work for slow release for a highly volatile component. Line 92-96

Materials and Method

1. If you have pictures of behavioral analysis and mortality of fish, please include. This will be a good reference for repeating this kind of experiment.
2. Explain the line 186-193 with the picture for each Stage I-III

Results

1. Table 2 and Table 3, please include in title the RR ratio index range based on WHO reference. Example; According to WHO RR < 5 indicates a susceptible field population; RR from 5-10 indicates moderate resistance and RR > 10 indicates high resistance, (Monitoring and managing insecticide resistance in Aedes mosquito populations. Interim guidance for entomologists. WHO/ZIKV/VC/16.1. Geneva: World Health Organization; 2016)
