# Supplementary material for: Larvicidal properties of terpenoid-based nanoemulsions against the dengue vector Aedes aegypti L. and their potential toxicity against non-target organism
Source: PLoS One. 2024 Feb 7;19(2):e0293124. doi: 10.1371/journal.pone.0293124 (PMC10849395; doi:10.1371/journal.pone.0293124)
Supplement: Attachment — Submitted filename: Response to Reviewers.docx [file pone.0293124.s003.docx]

We would like to express our sincere gratitude for the valuable feedback provided by the academic editor and reviewer(s) regarding our manuscript entitled " Larvicidal properties of terpenoid-based nanoemulsions against the Dengue vector Aedes aegypti L. and their potential toxicity against non-target organism." We appreciate their time and effort in thoroughly assessing our work. In response to their insightful comments, we have carefully considered each point raised and offer our rebuttal and revisions as follows:

**REVIEWER #1**

**Something seems wrong here. Why the total is not 100%?**

A: We corrected the information in the main text.

**What was the light exposure?**

A: We used a light/dark cycle of 12 hours. We added this information in the methods section.

**What is the meaning of AHI?**

A: We corrected the translation mistake in the main text. The correct acronym is IHC.

**One way ANOVA with N=5 do not seem reasonable. how the authors tested the normality of distribution?**

A: We used the Shapiro wilk test.

**Decide between BORA or Bora and correct it along the manuscript.**

A: Thank you for the suggestion. We standardized “Bora” throughout the text.

**Where is this data? refer it in the text please.**

A: We included a reference to this data in the text.

**The legend of this Table is surprisingly poor. It´s not possible to know what p (italics and lowercase, please) means, or which statistical test was performed, or even the number of biological replicas (n is number of mosquitoes?). Please provide explanation for this data and a better statistical report (use addiditonal tables if necessary).**

A: We reformulated the legend, adding the necessary information for data interpretation.

**Which test was used for the normality of distribution? ANOVA implies this condition.**

A: We used the Shapiro wilk test to check the normality of the data.

**Why GC?**

A: We corrected the translation mistake in the main text.

**Other strategies of stabilization of essential oils by encapsulation were alerady proposed, for example yeast encapsulation, as demonstrated in:**

**1. Parasit Vectors. 2021 May 22;14(1):272. doi: 10.1186/s13071-021-04733-2.**

**2. Parasit Vectors. 2020 Jan 13;13(1):19. doi: 10.1186/s13071-019-3870-4.**

A: We appreciate the suggestion. We would like to reiterate that the objective of this article is to discuss approaches related to nanoemulsions and larvicidal activity. At this time, we have chosen not to include other approaches in the manuscript.

**REVIEWER #2**

Introduction

Good explanations on the introduction about the mechanisms of resistant and objective of this study. Very clear and on the points for a research statements. The experiment design is much targeted and design well. Appropriate in this context.

**Line 56-65 can be mentioned more comprehensive, especially the alternative method. You are not using those method and not relevant in your study scope**

A: Thank you for the suggestion. We have chosen to remove that section from the manuscript.

**Explain more on the Nano emulsions technology for slow release. How the mechanisms work for slow release for a highly volatile component. Line 92-96**

A: We appreciate the reviewer's comment. We have included a detailed explanation on Nano emulsion technology for slow release in the manuscript.

Materials and Method

**If you have pictures of behavioral analysis and mortality of fish, please include. This will be a good reference for repeating this kind of experiment.**

A: Thank you for your suggestion. While we understand the value of including pictures of behavioral analysis and fish mortality in the manuscript, we would like to clarify that these specific visuals are not available in our study. However, we have provided detailed descriptions of the behavioral analysis and fish mortality results in the text. Additionally, we have referenced relevant literature that provides visual documentation of similar experiments, which can serve as a valuable reference for researchers interested in replicating these experiments.

**Explain the line 186-193 with the picture for each Stage I-III**

A:Thank you for your suggestion. While we understand the value of including pictures of behavioral analysis and fish mortality in the manuscript, we would like to clarify that these specific visuals are not available in our study.

Results

**Table 2 and Table 3, please include in title the RR ratio index range based on WHO reference. Example; According to WHO RR < 5 indicates a susceptible field population; RR from 5-10 indicates moderate resistance and RR > 10 indicates high resistance, (Monitoring and managing insecticide resistance in Aedes mosquito populations. Interim guidance for entomologists. WHO/ZIKV/VC/16.1. Geneva: World Health Organization; 2016)**

A: We included this information in the method section as this is not a result (P148-150).

We hope that these revisions adequately address the concerns raised and align the manuscript more closely with the objectives and scope of PLOS ONE. We are confident that the revised version now meets the high standards of academic excellence set by your esteemed journal.

Once again, we would like to express our sincere appreciation for the thorough review and constructive feedback provided by the academic editor and reviewer(s). Their expertise and guidance have undoubtedly improved the quality of our work.

Thank you for considering our revised manuscript. We look forward to receiving your final decision regarding its publication.

Yours sincerely,

Vincent Corbel
