# Supplementary material for: Larvicidal properties of terpenoid-based nanoemulsions against the dengue vector Aedes aegypti L. and their potential toxicity against non-target organism
Source: PLoS One. 2024 Feb 7;19(2):e0293124. doi: 10.1371/journal.pone.0293124 (PMC10849395; doi:10.1371/journal.pone.0293124)
Supplement: Attachment — Submitted filename: REVIEWER2.docx [file pone.0293124.s004.docx]

**REVIEWER #2**

Introduction

Good explanations on the introduction about the mechanisms of resistant and objective of this study. Very clear and on the points for a research statements. The experiment design is much targeted and design well. Appropriate in this context. Line 56-65 can be mentioned more comprehensive, especially the alternative method. You are not using those method and not relevant in your study scope

A: Thank you for the suggestion. We have chosen to remove that section from the manuscript. Explain more on the Nano emulsions technology for slow release. How the mechanisms work for slow release for a highly volatile component. Line 92-96

A: We appreciate the reviewer's comment. We have included a detailed explanation on Nano emulsion technology for slow release in the manuscript.

**Review: Acceptable and suitable**

Materials and Method

If you have pictures of behavioral analysis and mortality of fish, please include. This will be a good reference for repeating this kind of experiment.

A: Thank you for your suggestion. While we understand the value of including pictures of behavioral analysis and fish mortality in the manuscript, we would like to clarify that these specific visuals are not available in our study. However, we have provided detailed descriptions of the behavioral analysis and fish mortality results in the text. Additionally, we have referenced relevant literature that provides visual documentation of similar experiments, which can serve as a valuable reference for researchers interested in replicating these experiments. Explain the line 186-193 with the picture for each Stage I-III

A:Thank you for your suggestion. While we understand the value of including pictures of behavioral analysis and fish mortality in the manuscript, we would like to clarify that these specific visuals are not available in our study.

**Review: Acceptable and suitable**

Results

Table 2 and Table 3, please include in title the RR ratio index range based on WHO reference. Example; According to WHO RR < 5 indicates a susceptible field population; RR from 5-10 indicates moderate resistance and RR > 10 indicates high resistance, (Monitoring and managing insecticide resistance in Aedes mosquito populations. Interim guidance for entomologists. WHO/ZIKV/VC/16.1. Geneva: World Health Organization; 2016)

A: We included this information in the method section as this is not a result (P148-150).

**Review: Acceptable and suitable**
